# Health Policies to Control Chagas Disease Transmission in European Countries

**DOI:** 10.1371/journal.pntd.0003245

**Published:** 2014-10-30

**Authors:** Ana Requena-Méndez, Pere Albajar-Viñas, Andrea Angheben, Peter Chiodini, Joaquim Gascón, José Muñoz, Chagas Disease COHEMI Working Group

**Affiliations:** 1 Barcelona Centre for International Health Research (CRESIB, Hospital Clínic-Universitat de Barcelona), Barcelona, Spain; 2 Department of Control of Neglected Tropical Diseases, World Health Organization, Geneva, Switzerland; 3 Centre for Tropical Diseases (CTD), Sacro Cuore Hospital, Verona, Italy; 4 Hospital for Tropical Diseases London UK - National Institute for Health Research University College London Hospitals Biomedical Research Centre, London School of Hygiene and Tropical Medicine, London, United Kingdom; René Rachou Research Center, Fiocruz, Belo Horizonte, Brazil, Brazil

## Introduction

Chagas disease (CD) is a highly prevalent parasitic disease in immigrants from Mexico, as well as all of Central and South America. The total number of infected people is estimated between eight and ten million [1], [2], of whom 30%–40% either have, or will, develop cardiopathy, gastrointestinal disease, or both [1]. Cardiac involvement is the main cause of death from this infection through arrhythmias and cardiomyopathy. Nifurtimox and benznidazole are the only available medicines with proven efficacy against *Trypanosoma cruzi* infection in acute, congenital infection and early chronic infection. Until recently the treatment of chronic disease, particularly of adult patients with indeterminate form, was controversial; but during the past decade there has been a trend to offer treatment to adult patients and those with early cardiomyopathy [3].

To understand the magnitude of the problem, some economic studies have calculated the global cost of the disease worldwide at around 7,200,000,000 American dollars per year [4], which is mainly due to cardiovascular disease and early mortality. This cost is similar to, or even higher than, other prominent conditions such as rotavirus disease or cervical cancer [4].

In endemic countries, the main transmission route to humans is vectorial transmission through the faeces of infected triatomine bugs [1]. Oral transmission also occurs in endemic countries when beverages or food are contaminated with triatomine faeces [5]. Transmission through blood transfusion, organ transplantation from an infected donor, or from mother to child are less common routes, although they are of increasing importance, particularly in nonendemic areas where vectorial and oral transmission do not occur [1]. Another sporadic route of transmission is through the syringe sharing among drug users [6].

During the past decade, the infection has become a public health problem in some nonendemic countries, mainly due to migration and the chronic carriage of *T. cruzi* infection among a proportion of immigrants from endemic Latin American countries [7]. Since the first report of a case of CD in Europe was published in 1981 [8], sporadic cases have been detected in different European countries [9]. Since 2000, the number of reported cases has alarmingly increased, particularly in Spain and, to a lesser extent, in Italy and Switzerland [9]–[15].

In Europe, the currently estimated number of people with CD is between 68,000 and 122,000, but by 2009 only 4,290 had been diagnosed (index of underdiagnosis 93.9%–96.4%) [16].

The risk of transmission of *T. cruzi* infection in nonendemic countries through blood transfusion and organ transplantation has been described in multiple studies in the USA and more recently in Europe [17]–[21]. Moreover, several confirmed cases of *T. cruzi* transmission have already been detected in Europe [9], [22]. Accordingly, some studies have shown that it is cost-effective to screen for *T. cruzi* infection at blood banks, but depending on the prevalence of the disease, a mass screening of subjects—testing all—or a more selected strategy with screening questions to determine the risk level—screening and testing—should be applied [23].

Regarding congenital transmission, several studies have reported a rate of seroprevalence in Europe from 1.53% to 9.7% in pregnant women with the Bolivian population showing the highest seroprevalence rate [10], [24], [25], and with a transmission rate to newborn of around 7.3% [10].

Moreover, the strategy of screening pregnant women to control and treat newly diagnosed infected newborns has also shown to be cost-effective [26].

In response, several nongovernmental and later governmental initiatives have developed strategies to tackle this public health problem in the last years. The main aim of these initiatives has been to control the main transmission routes in nonendemic countries. Accordingly, some European countries have implemented national or regional measures to control transmission [27], [28], but many countries still have no legislation about it.

In 2009, the World Health Organization (WHO) convened a WHO informal consultation (jointly organized by WHO headquarters and the WHO Regional Office for Europe) that performed a comprehensive review outlining this specific issue in Europe [29]. In collaboration with WHO, a research group working on migrant health, COHEMI, (COordinating resources to assess and improve HEalth status of MIgrants from Latin America) has undertaken this study aimed at reviewing the health policies implemented in European Union countries with the highest disease prevalence, plus Switzerland, to control the transmission of CD through blood transfusion, organ transplantation, and the congenital route.

## Methods

A comprehensive search was performed to review health policies to control transmission of CD in different European countries. We selected countries belonging to the European Union (EU) before 2004. Switzerland was also included for the purpose of this study due to the high number of at risk immigrants as well as the increasing number of confirmed cases of *T. cruzi* infection in that country [30]. Countries joining the EU after 2004 were not included because of the low number of at risk immigrants and scarce or nonexistent data about CD in these countries.

**Figure 1:**
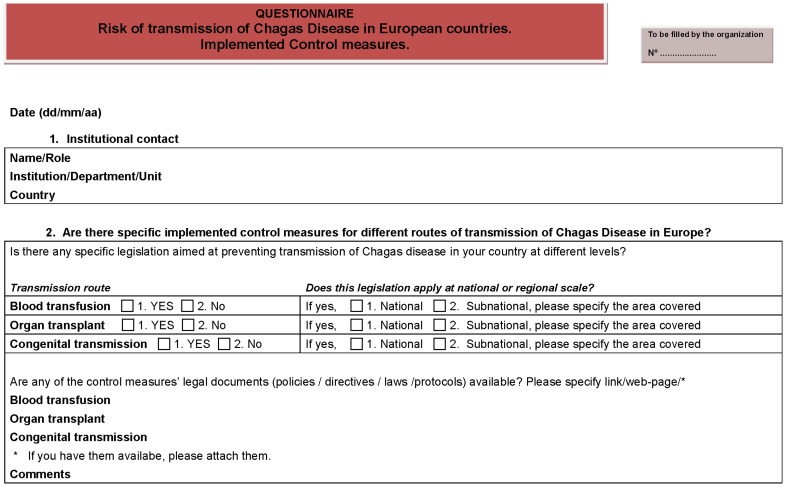
Questionnaire on health policy on *T. cruzi* infection in EU countries.

The search strategy, available at http://www.cohemi-project.eu/Pages/Our_Activities/Products/Other.aspx, was based on an online search of health policies in blood banks, transplant, and antenatal care programs. In addition, a short questionnaire was distributed among one or two experts in tropical medicine and international health from 15 European countries (Figure 1). Respondents were asked to provide information about existing policies implemented to control CD transmission in their countries or regions. They were also asked to provide the contact details of acting experts of the Ministry of Health in their countries and in the European Union as well as from blood banks, national transplant organizations, and antenatal care services of different European countries. When available, these experts and/or organizations were also contacted to confirm or to provide some specific information about a particular country. The information was validated when the directive, legislation, protocol, or document was obtained and accordingly, it was checked to see if the document contained a specific mention about how to control *T. cruzi* infection. The search was updated in September 2013. A map illustrating the measures currently implemented to control transmission in European countries (blood banks, organ transplantation, and congenital transmission) at the national and subnational levels was built.

## Results

A total of 15 questionnaires were received from 17 experts in 12 European countries. In addition, 18 blood banks from 14 countries and nine transplant organizations from seven EU/European Economic Area (EEA) countries were further contacted, and reply was received from nine blood banks and seven transplant organizations. No data were obtained from three countries (Austria, Greece, and Ireland). These three countries are estimated to host a low number of immigrants at risk and were not considered further in this study.

**Figure 2:**
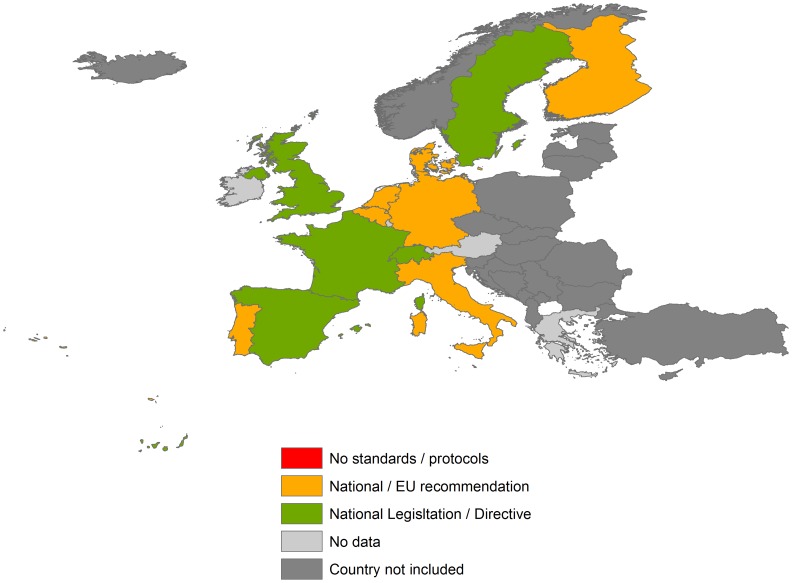
Blood transfusion health policy on *T. cruzi* infection in EU countries and Switzerland.

**Figure 3:**
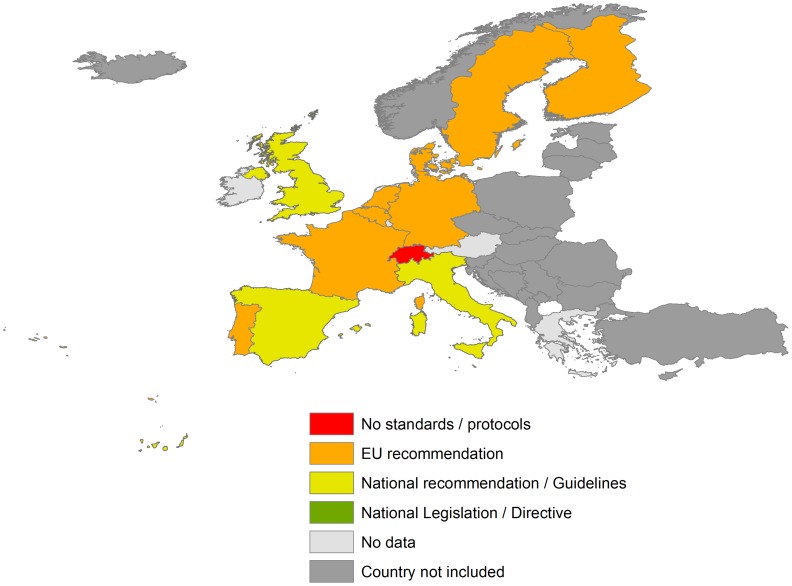
Transplantation health policy on *T. cruzi* infection in EU countries and Switzerland.

The data obtained are shown in Figures 1, 2, and 3 and summarized in the following paragraphs.

### Blood transfusion health policy

The United Kingdom has implemented systematic screening of at risk blood donations for *T. cruzi* infection since 1999 through professional guidelines issued by the Joint United Kingdom Blood Transfusion Services Professional Advisory Committee (JPAC). In Spain and France, obligatory screening has been implemented since 2005 and 2009 respectively in those people at risk of infection. Switzerland is the last country to have changed its directives in this regard (in January 2013) (Figure 2).

In these four countries, (i) blood donors born in endemic areas, or (ii) donors born to mothers native of endemic areas, or (iii) recipients of blood transfusions in endemic areas must be tested for *T. cruzi* infection before the blood donation. Italy is currently processing a similar change in the blood transfusion regulation in order to test all individuals at risk of *T. cruzi* infection, but the measure had not been officially approved at the time of the last update of the search strategy. In Portugal, a blood safety protocol will shortly be approved by the Instituto Português do Sangue e da Transplantação. Following this protocol, people at risk for *T. cruzi* infection will be directly excluded from donation. After the approval, a new directive needs to be implemented. In Sweden, systematic screening has not been implemented in blood banks, but all individuals who lived more than five years in Chagas disease–endemic countries (irrespective of whether or not they were born there) are systematically excluded from donation.

The other European countries are currently applying the European Commission's directives, 2004/33/CE and 2006/17/CE, approved by the EU related to quality and safety of blood, tissue, and cell donation in blood banks (Table 1). In these documents, individuals known to be infected with *T. cruzi* are specifically mentioned and defined as an exclusion criteria for blood donation. However, these directives do not specify which measures must be taken for those donors who were potentially exposed to *T. cruzi* infection in the past but who have not yet been tested. In contrast, a guideline from the Council of Europe entitled “Guide to preparation Use and Quality Assurance of Blood Components” (16th edition) specifically recommends performing a validated test for *T. cruzi* infection in donors who were born or transfused in areas where the disease is endemic (Table 1). Accordingly, the EU directive is out of step with the Council of Europe's recommendations. The authors of this paper did not find any source to determine whether all blood banks from any country without a specific national directive or legislation in this regard are following this recommendation.

**Table 1:** European or national directives, legislations, and guidelines to prevent transmission of Chagas disease.

	COUNTRY	DIRECTIVE	URL	YEAR
TRANSFUSSION	SPAIN	REAL DECRETO 1088/2005	http://www.boe.es/boe/dias/2005/09/20/pdfs/A31288-31304.pdf	2005
	FRANCE	Arrêtédu 12 janvier 2009: Legifrance, editor. *NOR SJSP0901086A*	http://www.legifrance.gouv.fr/affichTexte.do?cidTexte=JORFTEXT000020104647&dateTexte=&categorieLien=id	2009
	UNITED KINGDOM	Guidelines for the Blood Transfusion Services In: Services UBTTT. The Stationery Office (TSO)	https://www.gov.uk/government/uploads/system/uploads/attachment_data/file/228828/0117033715.pdf	2005
	SWEDEN	Socialstyrelsens föreskrifter om blodverksamhet;	http://www.socialstyrelsen.se/sosfs/2009-28/Documents/2009_28_rev.pdf	2009
	SUIZA	Prescriptions du Service de transfusion sanguine CRS	http://sbsc-bsd.ch/dokuman/Portals/0/kip/m0/1299/1305/21740-Aptitude-V08__8__8.pdf	2013
	EU	Commission Directive 2004/33/EC, 2004	http://eur-lex.europa.eu/LexUriServ/LexUriServ.do?uri=OJ:L:2004:091:0025:0039:EN:PDF	2004
	EU	Commission Directive 2006/17/EC, 2006	http://eur-lex.europa.eu/LexUriServ/LexUriServ.do?uri=OJ:L:2006:038:0040:0052:EN:PDF	2006
	COUNCIL OF EUROPE	Guide to the preparation, use and quality assurance of blood components. 16^th^ edition 2010	http://tots.edqm.eu/entry.htm	
	2010			
TRANSPLANT	EU	Directive 2010/45/EU	http://eur-lex.europa.eu/LexUriServ/LexUriServ.do?uri=OJ:L:2010:189:0001:0008:EN:PDF	2010
	SPAIN	Criterios de selección del donante de órganos respecto a la transmisión de infecciones	http://www.ont.es/infesp/DocumentosDeConsenso/infecciondonante.pdf	2004
	ITALY	Criteri generali per la valutazione di idoneità del donatore	http://www.sanfilipponeri.roma.it/trapianti/file/140812_linee_guida_donatore.pdf	2012
	UNITED KINGDOM	Guidance on the Microbioolgical safety of human organ, tissues and cells used in transplantation. SaBTO	https://www.gov.uk/government/publications/guidance-on-the-microbiological-safety-of-human-organs-tissues-and-cells-used-in-transplantation	2011
	COUNCIL OF EUROPE	Safety and quality assurance for the transplantation of organs, tissues and cells	http://tots.edqm.eu/entry.htm	2010
CONGENITAL	SPAIN (Catalonia)	Protocol de cribratge i diagnòstic de malaltia de Chagas en dones embarassades llatinoamericanes i en els seus nadons	http://www20.gencat.cat/docs/canalsalut/Home%20Canal%20Salut/Professionals/Temes_de_salut/Chagas/documents/Protocol_cribratge_Chagas_def.pdf	2010
	SPAIN (Valencia)	Enfermedad de Chagas importada protocolo de actuación en la comunitat Valenciana	http://publicaciones.san.gva.es/publicaciones/documentos/V-5243-2008.pdf	2008
	SPAIN (Galicia)	protocolo de cribado da enfermidade de Chagas en mulleres embarazadas	http://www.sergas.es/Publicaciones/DetallePublicacion.aspx?IdPaxina=60020&Idioma=es&IDCatalogo=2215	2012
	ITALY (Toscana)	Prevenzione e controllo della malattia di Chagas congenita: indicazioni per l'assistenza in gravidanza	http://parlamentosalute.osservatorioistituzioni.it/system/attachments/assets/000/007/568/original/489a.pdf	2012

### Transplantation health policy

We did not find any national directive or legislation regarding solid organ transplantation that includes Chagas disease as a specific topic. The EU directive concerning the regulation of solid organ donations does not specifically address measures to control *T. cruzi* infection. It mentions only that there are particular conditions that might affect the suitability of organs for transplantation because they imply the risk of infectious disease transmission.

Three national transplant organizations from Italy, Spain, and the United Kingdom have included a specific section regarding how to control transmission of Chagas disease through organ transplantation in their national guidelines about quality of safety of solid organ transplants (Figure 3). Accordingly, transplant donors in these countries are routinely screened, although seropositivity for Chagas disease might not be a contraindication for some type of transplants. By way of example, in Spain there is a very clear recommendation to test for *T. cruzi* in all donors at risk of infection; recipients of an organ from a positive donor are strictly followed up and monitored for the development of *T. cruzi*. Spanish and Latin American experts in Chagas disease have published recommendations concerning the management of Chagas disease in transplants [31]. In the UK, professional guidelines are produced by the Department of Health's Advisory Committee on the Safety of Blood, Tissues and Organs (SaBTO). The donor patient assessment form includes questions which permit an assessment of the donor possibly harbouring *T. cruzi* infection to be undertaken and testing to be performed where relevant.

The Council of Europe has also elaborated a guideline entitled “Safety and Quality assurance for the transplantation of organ, tissues and cells” (4th edition, 2010) (Table 1). This guideline also recommends testing donors from endemic areas and specifies criteria for transplant contraindications.

Information about Germany, France, or Finland, which recommends following the Council of Europe's guidelines, has been gathered, but the authors of this paper could not obtain any specific national document to corroborate this recommendation. To the best of our knowledge, the rest of the European countries apply EU or similar directives, but this paper has not assessed the degree to which the guidelines of the Council of Europe are applied.

### Congenital transmission

**Figure 4:**
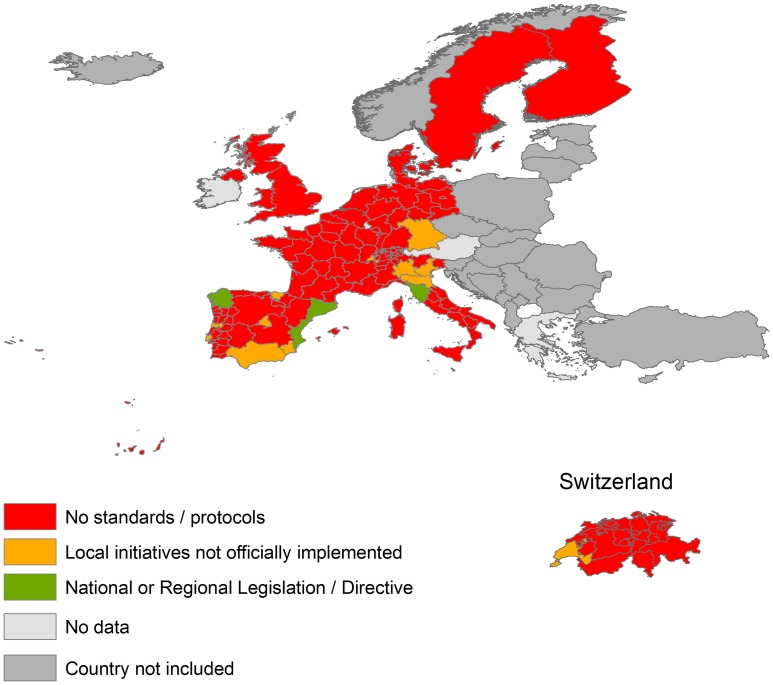
Control of congenital transmission of *T. cruzi* infection in EU countries and Switzerland.

European countries have no legislation requiring screening of pregnant women coming from Chagas disease–endemic areas and monitoring their offspring, except in three autonomous communities in Spain (Catalonia, Galicia, and Valencia) and one region in Italy (Tuscany) (Figure 4). In these four regions, a *T. cruzi* test must be done as a part of the required screening tests undertaken during antenatal care. When pregnant women test positive, newborns are tested for congenital Chagas disease and treated early if found to be infected with *T. cruzi* or followed up for at least nine months until the infection can be reliably ruled out. On the other hand, nongovernmental programmes are slowly being implemented in some other regions of Spain (four autonomic communities), Italy (three regions), Germany (one state), Portugal (two districts), and Switzerland (two cantons) (Figure 3). In these cases, protocols are being applied in some hospitals and maternity units but they cannot be considered as official recommendations. In the UK, there is no legislation and currently no routine antenatal screening programme for Chagas disease. However, the UK Health Protection Agency (now Public Health England) Migrant Health Guide 2011 states that pregnant women from at risk groups should be offered serological testing, and any positives should be referred to a specialist centre. Infants of seropositive mothers should be followed up to detect and treat any cases of vertical transmission. In the remaining countries there is no legislation concerning Chagas disease.

## Discussion

Transmission of CD is a real possibility in countries hosting populations from Latin American endemic countries [9], [10], [18], [30]. Some European countries, particularly those with a large number of Latin American immigrants, are slowly acknowledging this growing public health problem, and some changes in health policies have been made.

With reference to transmission through blood transfusion, seven countries have either already implemented, or are in the process of, changing their recommendations to enhance detection of cases of *T. cruzi* infection: France, Italy, Portugal, Spain, Sweden, Switzerland, and the United Kingdom. These countries have taken the initiative to change their practice; they have the highest percentage of Latin American immigrants and the highest risk of transmitting the disease. In Sweden, where systematic screening has not been implemented in blood banks, but all individuals who lived more than five years in Chagas disease–endemic countries are systematically excluded from donation, the risk of disease transmission through transfusion is substantially reduced, but gaps remain in this regulation such as in the case of donors whose mothers were born in endemic areas. For the rest of the European countries, the official directives either exclude donations only in patients confirmed to be infected, or do not apply any measure. The problem is that the majority of chronically infected people either do not show any symptoms or have symptoms that simulate other conditions; therefore, the possibility of CD might not be suspected. When such people are not screened, there is a potential risk of transmission of *T. cruzi* infection in those countries. Furthermore, because of the particularities of CD, a deferral period in blood donation six months after the migrant process or after the last trip, which is often applied, does not give any protection against transmission of Chagas disease in transfusional practice.

A European guideline recommends screening all people at risk of Chagas disease, but we do not know if national blood banks are applying it since the extent to which it has been implemented has not been assessed. Even though the total number of Latin American immigrants in these countries is low compared to the seven countries mentioned, potential transmission of Chagas disease is a public health concern. This problem might become more relevant in the context of the recent economic crisis given the unpredictable internal migration flows within European countries. It must also be considered that some of these immigrants might have received a European nationality at the time of their re-migration, making it more difficult to identify individuals at risk of the infection.

With regards to health policies for solid organ transplantation, no country has a specific directive or legislation that considers Chagas disease in all patients at risk. Even the EU directives do not specifically mention *T. cruzi* infection as a potential risk for recipients of solid organ transplants. In such cases, the severity of the disease is potentially more important because these patients will be permanently immunosuppressed.

Although the Council of Europe guideline mentions Chagas disease risk and is theoretically followed by some European countries, we do not know if testing has been systematically implemented by national transplant programmes. To our knowledge, only three countries (Italy, Spain, and the United Kingdom) have national guidelines to control this route of transmission through systematic screening in all donors at risk of the infection. The outlook is even less promising concerning the congenital transmission route. Only four first administrative divisions (FAD) among countries considered have implemented adequate policies to test newborns at risk of the infection. As far as we know, the rest of FAD and other countries have not any protocols or directives with the exception of some non-official local initiatives in some FAD. Control of congenital transmission has been demonstrated to be one of the most cost-effective measures to control the disease [26]. Since newborns with acute disease can be cured easily if diagnosed and treated early, subsequent cardiac, digestive, and neurological complications might be prevented in a significant percentage of affected people, and the possibility of further transmission to subsequent generations will also be avoided.

This study has several limitations. First, other non-official initiatives to control the transmission of the infection might exist but we could not find them. Moreover, in countries where a directive already exists, we have not evaluated the correct implementation of the directive or policy. Thus, we cannot be sure that the transmission of the infection is avoided. Finally, not all European countries were included in the study; however we should also consider that most Latin American immigrants in Europe are living in the European countries that were described in our study.

## Conclusions and Recommendations

To ensure control of Chagas disease transmission in European countries, changes in some laws and directives concerning blood banks and transplant programmes are urgently needed to avoid or reduce the risk of transmission. It would be useful that regulations emanating from the European Commission were in accordance with the recommendations from the Council of Europe. In addition, in countries where legislation exists, efforts should be made to evaluate the implementation of these directives in blood banks to apply adequate systematic screening of all people at risk of *T. cruzi* infection with a validated method, incorporating internal and external quality controls of the serological test. Finally, programmes to control congenital Chagas disease should be implemented in all European countries based on the screening of pregnant women at risk of the infection, with the primary objective of treating infected newborns at an early stage.
